# Supplementary material for: Acute cerebellar knockdown of Sgce reproduces salient features of myoclonus-dystonia (DYT11) in mice
Source: eLife. 2019 Dec 23;8:e52101. doi: 10.7554/eLife.52101 (PMC6959989; doi:10.7554/eLife.52101)
Supplement: Transparent reporting form [file elife-52101-transrepform.pdf]

eLIFE
1st Floor
24 Hills Road
Cambridge CB2 1JP, UK
P 01223 855340
W elifesciences.org
T @elife

eLife's transparent reporting form

We encourage authors to provide detailed information *within their submission* to facilitate the interpretation and replication of experiments. Authors can upload supporting documentation to indicate the use of appropriate reporting guidelines for health-related research (see EQUATOR Network), life science research (see the BioSharing Information Resource), or the ARRIVE guidelines for reporting work involving animal research. Where applicable, authors should refer to any relevant reporting standards documents in this form.

If you have any questions, please consult our Journal Policies and/or contact us: editorial@elifesciences.org.

Sample-size estimation

- You should state whether an appropriate sample size was computed when the study was being designed
- You should state the statistical method of sample size computation and any required assumptions
- If no explicit power analysis was used, you should describe how you decided what sample (replicate) size (number) to use

Please outline where this information can be found within the submission (e.g., sections or figure legends), or explain why this information doesn't apply to your submission:

We did not perform a power analysis prior to start of the experiments. This is because, based on past experience with these types of experiments (when we generated comparable mouse models of two other dystonias, DYT1 and DYT12), we knew that in addition to biological variability a number of additional factors contribute to the variability of symptoms in mice. These include the extent to which from injection to injection the virus spreads, how well the cells are transfected, and how much the protein is knockdown in each animal. To overcome these sources of variability we overpowered our studies by using a large number of animals and many repetitions. Throughout the text and figure legends we have noted the number of animals used.

Replicates

- You should report how often each experiment was performed
- You should include a definition of biological versus technical replication
- The data obtained should be provided and sufficient information should be provided to indicate the number of independent biological and/or technical replicates
- If you encountered any outliers, you should describe how these were handled
- Criteria for exclusion/inclusion of data should be clearly stated
- High-throughput sequence data should be uploaded before submission, with a private link for reviewers provided (these are available from both GEO and ArrayExpress)

Please outline where this information can be found within the submission (e.g., sections or figure legends), or explain why this information doesn't apply to your submission:

eLife Sciences Publications, Ltd is a limited liability non-profit non-stock corporation incorporated in the State of Delaware, USA, with company number 5030732, and is registered in the UK with company number FC030576 and branch number BR015634 at the address 1st Floor, 24 Hills Road, Cambridge CB2 1JP | August 2014

Biological replicates in these experiments are individual mice while technical replicates are cohorts of mice injected at the same time. Data from cohorts of mice were found to be comparable.

The number of animals is indicated with "N" and is explicitly stated in the text after each experiment, as well as in the figure legends.

With three exceptions as outlined below, all data were included in the analysis and are reported, and individual data points are shown whenever possible.

The exceptions are as follows:
1) On rare occasions, animals were excluded when there was no clear GFP expression in the cerebellum or basal ganglia, suggesting that the virus, which encodes a GFP reporter, was not effectively injected into the brain, likely due to a clogged syringe during surgery.
2) Animals were excluded if post-hoc histology revealed expression of the virus outside the cerebellum or basal ganglia, when each were targeted respectively.
3) Toward the end of our studies, we run out of antibody and received a new batch. This batch of antibody was suspect, and did not seem to specifically label the protein. As a result, we excluded a limited number of experiments which were conducted with this second batch of antibody.

Statistical reporting

- Statistical analysis methods should be described and justified
- Raw data should be presented in figures whenever informative to do so (typically when N per group is less than 10)
- For each experiment, you should identify the statistical tests used, exact values of N, definitions of center, methods of multiple test correction, and dispersion and precision measures (e.g., mean, median, SD, SEM, confidence intervals; and, for the major substantive results, a measure of effect size (e.g., Pearson's r, Cohen's d)
- Report exact p-values wherever possible alongside the summary statistics and 95% confidence intervals. These should be reported for all key questions and not only when the p-value is less than 0.05.

Please outline where this information can be found within the submission (e.g., sections or figure legends), or explain why this information doesn't apply to your submission:

Statistical methods are reported in the Methods section. Individual statistical tests are reported with the results of each experiment.

(For large datasets, or papers with a very large number of statistical tests, you may upload a single table file with tests, Ns, etc., with reference to sections in the manuscript.)

Group allocation

- Indicate how samples were allocated into experimental groups (in the case of clinical studies, please specify allocation to treatment method); if randomization was used, please also state if restricted randomization was applied
- Indicate if masking was used during group allocation, data collection and/or data analysis

Please outline where this information can be found within the submission (e.g., sections or figure legends), or explain why this information doesn't apply to your submission:

Mice were randomly assigned to experimental groups. Whenever possible, littermate controls were injected with NT shRNA on the same day that other mice were injected with shRNA against sgce.

Scoring of videos was performed by independent scores blinded to the experimental condition of the animal, as indicated in the text and methods.

Additional data files ("source data")

- We encourage you to upload relevant additional data files, such as numerical data that are represented as a graph in a figure, or as a summary table
- Where provided, these should be in the most useful format, and they can be uploaded as "Source data" files linked to a main figure or table
- Include model definition files including the full list of parameters used
- Include code used for data analysis (e.g., R, MatLab)
- Avoid stating that data files are "available upon request"

Please indicate the figures or tables for which source data files have been provided:

As much as possible, individual data are presented in the paper. Additional data files are, thus, not provided.
